# The HSP-RTK-Akt axis mediates acquired resistance to Ganetespib in HER2-positive breast cancer

**DOI:** 10.1038/s41419-021-03414-3

**Published:** 2021-01-26

**Authors:** Christopher E. Eyermann, John D. Haley, Evguenia M. Alexandrova

**Affiliations:** Department of Pathology, Stony Brook University, Stony Brook, NY 11794-8691 USA

**Keywords:** Breast cancer, Cancer therapeutic resistance

## Abstract

Breast cancer is the leading cause of cancer-related death in women worldwide. Human epidermal growth factor receptor 2 (HER2)-positive subtype comprises 20% of sporadic breast cancers and is an aggressive disease. While targeted therapies have greatly improved its management, primary and acquired resistance remain a major roadblock to making it a curable malignancy. Ganetespib, an Hsp90 (Heat shock protein 90) small molecule inhibitor, shows preferential efficacy in HER2-positive breast cancer, including therapy-refractory cases, and has an excellent safety profile in ongoing clinical trials (38 in total, six on breast cancer). However, Ganetespib itself evokes acquired resistance, which is a significant obstacle to its clinical advancement. Here, we show that Ganetespib potently, albeit temporarily, suppresses HER2-positive breast cancer in genetic mouse models, but the animals eventually succumb via acquired resistance. We found that Ganetespib-resistant tumors upregulate several compensatory HSPs, as well as a wide network of phospho-activated receptor tyrosine kinases (RTKs), many of which are HSP clients. Downstream of p-RTKs, the MAPK pathway remains suppressed in the resistant tumors, as is HER2 itself. In contrast, the p-RTK effector Akt is stabilized and phospho-activated. Notably, pharmacological inhibition of Akt significantly delays acquired Ganetespib resistance, by 50%. These data establish Akt as a unifying actionable node downstream of the broadly upregulated HSP/p-RTK resistance program and suggests that Akt co-targeting with Ganetespib may be a superior therapeutic strategy in the clinic.

## Introduction

Breast cancer is the most common female malignancy and the major cause of cancer-related mortality in women worldwide^1^. Human epidermal growth factor receptor 2 (HER2)-positive breast cancer comprises about 20% of all breast cancers and is defined by HER2 gene amplification/protein upregulation^2–5^. HER2 overexpression is a significant negative predictor of overall survival and time to relapse, due to enhanced cell proliferation, angiogenesis and metastasis, and reduced apoptosis^6–8^. Moreover, this molecular subtype is more frequently diagnosed in younger patients (median 57 vs. 64 y.o., METABRIC^9^) and at advanced metastatic stage III/IV, in 54% of cases compared with 36% of hormone receptor-positive/HER2-negative breast cancer and 41% of triple-negative breast cancer, another aggressive subtype^5^.

HER2-targeted therapies (Trastuzumab, Lapatinib) have greatly improved the treatment of HER2-positive breast cancer^4^. However, 36–76% patients show primary resistance to Trastuzumab, and the majority of initial responders progress within one year^10,11^. The search for alternative vulnerabilities revealed that the HER2 oncogene heavily relies on the heat shock protein 90 (Hsp90) molecular chaperone and is very sensitive to Hsp90 inhibitors^12–14^. Hsp90, one of the most abundant proteins in the cell, regulates normal cellular homeostasis by maintaining proper folding, stability and activity of its “client” proteins^15,16^. During tumorigenesis, Hsp90 is further upregulated in response to the proteotoxic stress and thereby confers superior proliferative, survival, angiogenic and metastatic properties to cancer cells^17,18^. Notably, of over 700 identified Hsp90 clients (www.picard.ch/downloads/Hsp90interactors.pdf), many are oncoproteins, including oncogenic drivers of breast cancer (e.g., estrogen and progesterone receptors, BRCA1/2), components of the HER2 signaling pathway (HER2, its co-receptor EGFR, downstream effectors Raf1, Erk, Akt, mTOR), and common mediators of therapeutic resistance in breast and other cancers (e.g., mutant HER2 and EGFR, HER3, IGF-1R)^12–15,19–23^.

Although Hsp90 inhibitors intercept multiple oncogenic and resistance pathways at once^18,24^, their clinical activity to date has been restricted to the cancers driven by the most sensitive Hsp90 clients, e.g., HER2-positive breast cancer^25–27^ and EML4-ALK-positive non-small-cell lung cancer^28,29^. Indeed, multiple first-generation Hsp90 inhibitors showed promising results in HER2-positive breast cancer clinical trials, but their development was stalled due to significant adverse effects, in particular liver and ocular toxicities^13^. In turn, a next-generation Hsp90 inhibitor Ganetespib (STA-9090, Synta Pharmaceuticals) emerged as a significantly safer alternative^25,26^ that has so far been evaluated in 38 clinical trials (two of them Phase III), including six on breast cancer. Of the latter, three completed studies strongly point to Ganetespib’s preferential efficacy in the HER2 subtype^25–27^. First, in the patients with metastatic breast cancer of all molecular subtypes, Ganetespib produced positive responses primarily in the HER2 group^25^. Second, Ganetespib—in combination with Trastuzumab and Paclitaxel—tested specifically in the HER2-positive breast cancer patients again showed a positive response and excellent safety profile^26^. Finally, a retrospective search for biomarkers of sensitivity to Hsp90 inhibitors found that HER2 is the most important—“and perhaps the only”—such biomarker^27^.

Despite Ganetespib’s great promise for HER2-positive breast cancer, patients in the clinical trials eventually progress on it, i.e., develop therapeutic resistance^25,26^. Therefore, the understanding and pharmacological interception of acquired Ganetespib resistance is critical for its clinical advancement and will also advance our knowledge on the entire class of Hsp90 inhibitors (none is FDA approved so far). We recently reported that Ganetespib is effective in a genetic mouse model of HER2-positive breast cancer after acquiring Lapatinib resistance, thus mimicking its activity in human therapy-refractory disease^30^. Therefore, genetic models are both appropriate and the most pre-clinically advanced to study this drug. Here, we tested the long-term effectiveness of Ganetespib in mouse models and found that, similar to patients, it evokes acquired resistance. Therefore, we set out to gain an in-depth understanding of the molecular mechanisms of acquired Ganetespib resistance in vivo, with the ultimate goal of advancing treatment options and improving outcomes for patients with HER2-positive breast cancer.

## Materials and methods

### **M**ouse strains, mammary fat pad transplantation, drug treatments

MMTV-Neu mice (strain FVB/N-Tg(MMTVneu)202Mul/J) and MMTV-ErbB2 mice (strain FVB/N-Tg(MMTV-ERBB2)NK1Mul/J) were from the Jackson Laboratory and were previously described^30–32^. Only females were used, since breast cancer is 100 times more prevalent in females than in males. Mice were treated with Ganetespib when autochthonous tumors appeared, i.e., at the age of 6−18 months. Immunodeficient athymic nude mice were from the Jackson Laboratory (strain Foxn1^nu/^/Foxn1^nu^, Bar Harbor, ME). For mammary fat pad transplantation of primary Neu tumor cells, tumor cells were isolated as described below. Freshly isolated tumor cells were injected into mammary glands #4 and #9 of 5 weeks old Foxn1^nu/^/Foxn1^nu^ females at 10,000 cells per injection, in 100 µl of 3:1 DMEM:Matrigel (Corning, #356234, Corning, NY). Autochthonous and allografted mammary tumors were measured weekly by caliper, tumor volume was calculated as l*w*h/2, where “*l*” is length, “*w*” is width, “*h*” is height (an approximate formula for the volume of ellipsoid). Ganetespib (Selleckchem, #S1159, Houston, TX) in Cremophor vehicle (10% DMSO/18% Cremophor/3.6% dextrose) was injected via tail vein at 80 mg/kg once a week. MK-2206 (Selleckchem, #S1078, Houston, TX) in Captisol vehicle (30% in water) was injected via oral gavage at 20 mg/kg three times a week. Treatments of the allografted animals started when the allografts reached ~90 mm^3^. All animals were treated humanely and according to the guidelines by the Stony Brook University Institutional Animal Care and Use Committee (IACUC). Sample size was not pre-determined, animals were assigned to treatment groups randomly, no blinding was used.

### **I**solation of primary Neu tumor cells for mammary gland allografts

Neu tumors were dissected and processed into single tumor cell isolates as previously described^33^. Tumor cells were grown in DMEM/F12 Medium (Gibco, #10565-018, Gaithersburg, MD) supplemented with 10% FBS for 2 weeks in a humidified chamber at 37 °C with 5% CO_2_. Tumor cells were detached from plates using accutase (Millipore Sigma, #SCR005, Burlington, MA), resuspended in CNT-Prime epithelial culture medium (CELLnTEC, #Cnt-PR, Bern, Switzerland), and incubated with EpCAM-APC (Invitrogen, #17-5791-82, Carlsbad, CA) and ErbB2/HER2-Alexa Fluor 594 (R&D Systems, #FAB6744T, Minneapolis, MN). The cells were sorted with a FACSAria III Cell Sorter (BD Biosciences, San Jose, CA) and analyzed using Cyflogic software (v1.2.1, CyFlo Ltd, Turku, Finland). The EpCAM-APC and ErbB2/HER2-Alexa Flour 594 double-positive cells were immediately used for transplantation.

### **Q**uantitative real-time reverse transcription polymerase chain reaction (RT-PCR)

Total RNA was extracted from the mammary tumors using the RNeasy Mini Kit (Qiagen, Hilden, Germany). Complementary DNA (cDNA) was prepared from 1 µg total RNA using random primers and SuperScript II reverse transcriptase (Invitrogen, Carlsbad, CA). The PCR primers were the following: Hsp90A ttgcttcagtgtcctggtg (F), cctgtttgctgggaatgag (R); Hsp90B cctgctctgtactactactc (F), aatgcctgtgtccaccaaag (R); Hsp70 gatcatcgccaacgaccag (F), ctcgcccttgtagttcacc (R); Hsc70 tggcattgatctcggcacc (F), acgcccgatcagacgtttg (R), Hsp27 cacagtgaagaccaaggaag (F), cctcgaaagtaaccggaatg (R), Hsp40 (aka Dnaja1, DnaJ) aaaacccaatgccacccag (F), tccatgggtgagccaaaac (R). Real-time PCR was performed using QuantiTect SYBR Green PCR kit (Qiagen, Valencia, CA) on an Applied Biosystems QuantStudio 3 Real-time PCR system and analyzed with QuantStudio Design & Analysis software version 1.4 (Applied Biosystems, Waltham, MA). The PCR conditions were: 94 °C for 30 s, 60 °C for 1 min and 72 °C for 1.5 min, 40 cycles. Samples were analyzed in duplicates and normalized to *HPRT* and to the mean signal for each gene.

### **W**estern blot analysis and phospho-RTK array assay

Mammary tumors were minced and resuspended in RIPA buffer (150 mM NaCl, 1% NP-40, 0.5% Deoxycholate, 0.1% SDS and 50 mM Tris HCl, pH 8) with protease inhibitors, sonicated, spun down, and fat was removed before protein quantification. Immunoblots were performed using 20–60 μg of protein using the following antibodies, all from Cell Signaling Technology (Danvers, MA): HER2 (#4290T), p-HER2 (Y1248, #2247T), p-HER2 (Y1221/1222, #2243T), Akt (#4691T), p-Akt (S473, #4060T), p-Akt (T308, #13038T), actin (#3700T). The mouse phospho-RTK analysis was performed using the Proteome Profiler Mouse Phospho-RTK Array Kit (R&D Systems, #ARY014, Minneapolis, MN) according to the manufacturer’s instructions. Signal densities were quantified using NIH Image software and normalized as indicated in the figure legends and also, to the background.

### **M**ass spectrometry

Control, Resistant-3 h and Resistant-24 h tumor samples from Neu mice that acquired Ganetespib resistance in vivo during at least 2 months were analyzed. Each sample contained three biological replicas pooled together. The samples in 8 M urea, 100 mM ammonium bicarbonate, were subjected to reduction (5 mM DTT), alkylation (10 mM iodoacetamide), diluted to 2 M urea and digested with trypsin at 37 °C overnight. Peptides were desalted, lyophilized, resuspended in 0.1% TFA, 50% acetonitrile, 1 M lactic acid and incubated with TiO_2_ beads for one hour. Peptides not binding to TiO_2_ beads with collected as a non-phosphorylated, total protein fraction, lyophilized and desalted. Peptides bound to TiO2 were washed with 0.1% TFA, 50% acetonitrile and eluted with 50 mM KH2PO4 pH 10.5, neutralized with 5% formic acid, 50% acetonitrile, lyophilized and desalted. Phospho-peptides and non-phosphorylated (total protein) peptides were both analyzed by liquid chromatography-electrospray ionization tandem mass spectrometry (LC-MS/MS), using orbital trap (Q-Exactive HF; ThermoFisher Scientific, Waltham, MA) and orthogonal quadruple TOF (5600Plus; Sciex, Framingham, MA) instruments followed by protein database searching. HPLC C18 columns were prepared using a P-2000 CO2 laser puller (Sutter Instruments, Novato, CA) and silica tubing (75 µm ID x ~10 cm) and were self-packed with 3 µm Magic AQ C18 resin. Protein abundance and peptide phosphorylation site abundance were established by protein database searching using the ProteomeDiscoverer v2.3 and ProteinPilot v5.01, followed by statistical analysis using JMP12. Three missed tryptic cleavages were allowed, and the posttranslational modifications considered included Ser, Thr and Tyr phosphorylation. Database searches used the mouse UniProt FASTA database (16982 reviewed sequences including common contaminants). False discovery rates of peptide capture experiments were typically <1%.

### **S**tatistical analysis

Appropriate statistical tests were used for each dataset, as follows. Mouse overall survival and progression-free survival was analyzed by Kaplan–Meier analysis and Log Rank statistics, the *p*-values and hazard rates were determined using an online software (www.evanmiller.org/ab-testing/survival-curves.html). Tumor size and mRNA and protein expression levels were analyzed by unpaired two-tailed Student’s *t*-test. *p* < 0.05 was considered statistically significant. No blinding was used. Estimate of variation was not performed, normal distribution was not tested for.

## Results

### **G**anetespib is effective against HER2-positive breast cancer in vivo, but eventually evokes acquired resistance

**Fig. 1:**
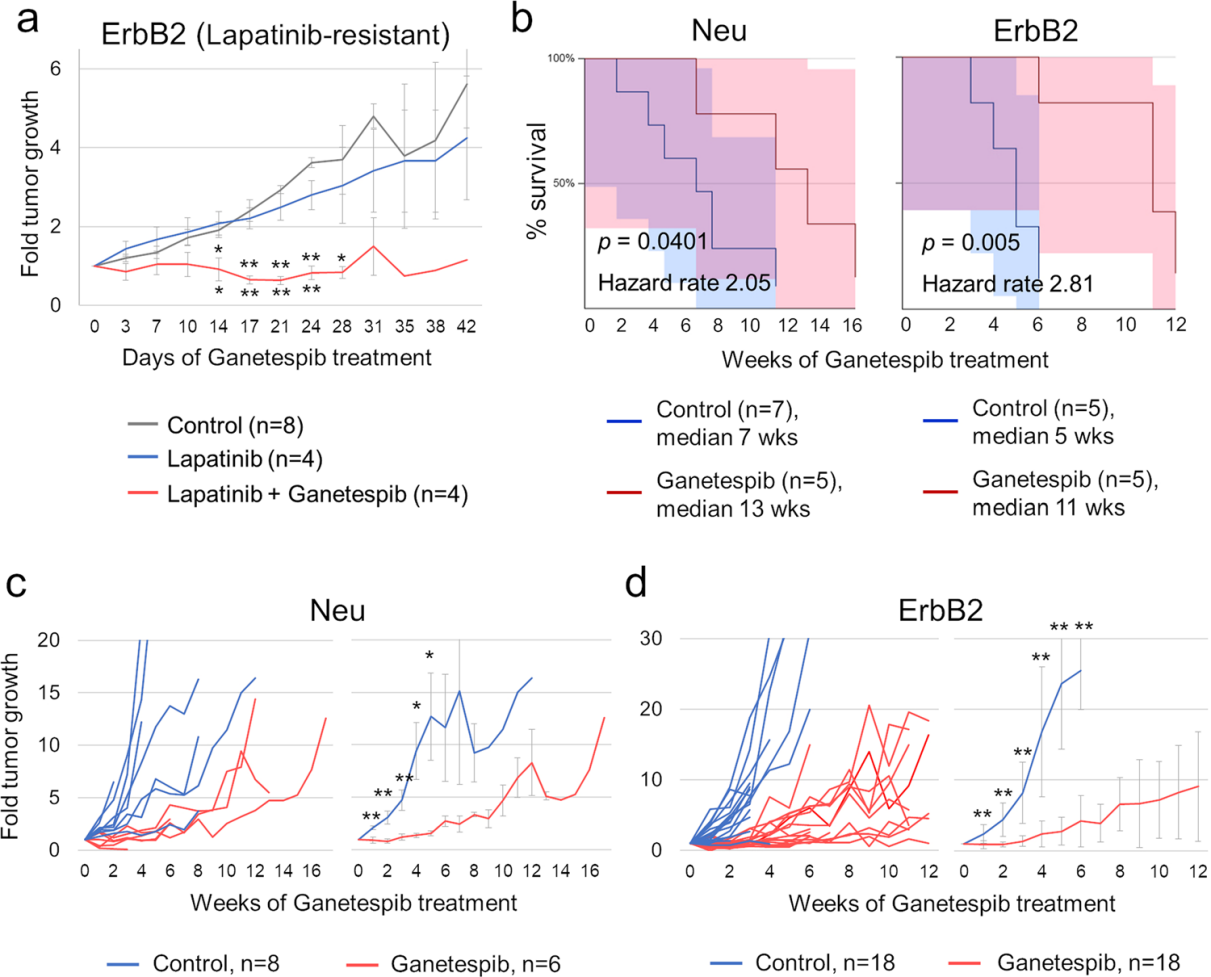
Ganetespib is effective in mouse models of HER2-positive breast cancer, but eventually evokes acquired resistance. **a** Tumor volume over time in Lapatinib-resistant ErbB2 mice. After acquiring Lapatinib resistance, mice were treated with Vehicles, Lapatinib alone (which was no longer effective), or Lapatinib and Ganetespib together. Tumor size was normalized to the respective initial size (when treatments started), which was on average 2,501 ± 156 mm^3^. Mean ± SEM, *p < 0.05, **p < 0.01 (top asterisks, vs. Vehicles; bottom asterisks, vs. Lapatinib alone), unpaired two-tailed Student’s *t*-test. **b** Overall survival of Neu (*left*) and ErbB2 (*right*) mice with or without (Control) Ganetespib injections, weeks after the onset of the autochthonous mammary tumors (when treatments started). Note that Ganetespib approximately doubles the overall survival in both models. Kaplan–Meier analysis, *p*, Log Rank statistics. Shaded areas are 95% confidence interval. **c**, **d** The dynamics of tumor growth over time in Neu (**c**) and ErbB2 (**d**) mice with or without (Control) Ganetespib injections performed as in **b**. Note that after the initial period of regression/stagnation for about 4 weeks, tumors in both models regrow due to acquired resistance. Tumor size was normalized to their respective initial size (when treatments started), which was 233 ± 22 mm^3^ and 251 ± 23 mm^3^ (**c**); 157 ± 45 mm^3^ and 146 ± 40 mm^3^ (**d**) for Control and Ganetespib groups, respectively. **c**, **d**, *left* growth of individual tumors, **c**, **d**, *right* average tumor growth. Mean ± SEM, *p < 0.05, **p < 0.01, unpaired two-tailed Student’s *t*-test.

While investigating acquired resistance to Lapatinib, we recently reported that Ganetespib potently suppresses HER2-positive breast tumors in a genetic mouse model, ErbB2;mutant p53 mice^30^. This is in agreement with clinical trials on patients with Trastuzumab/Lapatinib-resistant disease^25,26^, the majority of whom (about 70%) also carry mutant TP53^30^. To extend these studies, we then analyzed Ganetespib in p53-wildtype Lapatinib-resistant ErbB2 mice and found that it was also effective (Fig. 1a). This once again illustrates that HER2 is a much more sensitive Hsp90 client than many others, e.g., mutant p53^27,34^. We then tested the long-term activity of Ganetespib in two different genetic mouse models of HER2-positive breast cancer, ErbB2 and Neu mice^31,32^. Both of these models are widely used and represent the two main etiologies of HER2-positive breast cancer in humans: constitutively active HER2 (ErbB2 mice^32^) and amplified wild-type HER2 (Neu mice^31^), which is found in the majority of human cases^2–4^. We found that Ganetespib treatment significantly prolonged—approximately doubled—the overall survival of both cohorts (Fig. 1b). Notably, however, both cohorts showed tumor regression/stagnation for an average of 4 weeks (Fig. 1c, d), after which the tumors started to regrow due to acquired Ganetespib resistance. We used these resistant tumors to interrogate the in vivo resistance mechanisms.

### **U**pregulation of compensatory heat shock proteins in Ganetespib-resistant tumors

**Fig. 2:**
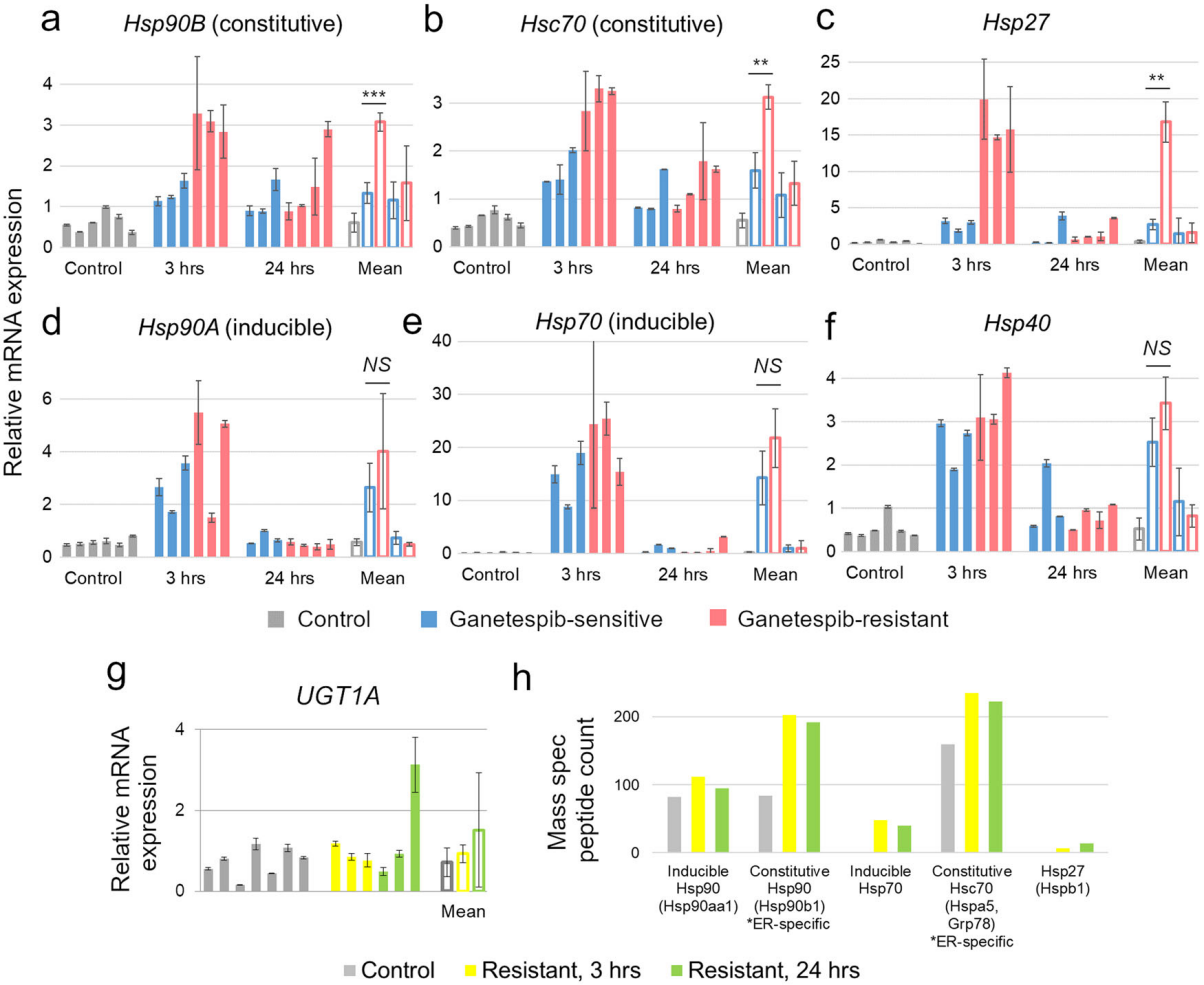
Upregulation of compensatory heat shock proteins (HSPs) in Ganetespib-resistant Neu tumors. **a**–**f** All analyzed HSPs are upregulated in Ganetespib-resistant and Ganetespib-sensitive tumors at 3 h after the last Ganetespib dose and decline at 24 h. However, constitutive *Hsp90B*, *Hsc70* and inducible *Hsp27* (**a–c**), but not inducible *Hsp90A*, *Hsp70* and co-chaperone *Hsp40* (**d–f**), are significantly more upregulated at 3 h in Ganetespib-resistant tumors, that had received Ganetespib for over two months, compared with Ganetespib-sensitive tumors, that received Ganetespib only once. **g** *UGT1A*, previously implicated in primary—not acquired—Ganetespib resistance in colorectal cancer, is not upregulated in Ganetespib-resistant Neu tumors. **a–g** Quantitative RT-PCR. mRNA levels were normalized to *HPRT*. Mean ± SD of at least three biological replicas (*empty bars*) and two technical replicas (*solid bars*) are shown. **p < 0.01, ***p < 0.001, *NS*, not significant; unpaired two-tailed Student’s *t*-test on the biological replicas. **h** Protein expression of the indicated HSPs at the indicated timepoints by mass spectrometry. Note that all HSPs are upregulated at both, 3 h and 24 h timepoints.

One predicted mechanism of therapeutic resistance to Hsp90 inhibitors is compensatory activation of alternative heat shock proteins by HSF1 (heat shock factor 1) transcription factor^35–37^. In naïve cells, Hsp90-bound HSF1 is sequestered in the cytoplasm, whereas Hsp90 inhibition activates HSF1, resulting in its nuclear translocation and a quick and transient transactivation of its *HSP* targets, so called inducible *Hsp90A, Hsp70*, and *Hsp27*^35–37^. Therefore, we hypothesized that Ganetespib resistance is mediated by upregulation of these or other HSPs and, consequently, stabilization of their oncogenic clients. Focusing on the Neu mouse model, since it better represents the most common etiology of the human disease^2–4^, we collected mammary tumors from Ganetespib-resistant and control mice at two different timepoints after the last Ganetespib dose (3 h and 24 h) and assessed the expression levels of various *HSPs* by quantitative RT-PCR (Fig. 2a–f). We used two types of negative controls: (1) untreated tumors (Control) and (2) Ganetespib-sensitive tumors that received Ganetespib only once, which would allow us to “subtract” Ganetespib’s short-term effects from the long-term resistance mechanisms. Not surprisingly, we found upregulation of all inducible *HSPs* that were tested, *Hsp90A*, *Hsp70*, *Hsp27* and their co-factor *Hsp40*, at 3 h post-last Ganetespib dose and their decline at 24 h (Fig. 2c–f). Surprisingly, however, we also found a significant upregulation of so called constitutive *HSPs*, *Hsp90B* and *Hsc70*, whose transcription is driven by core transcription factors (Fig. 2a, b). Moreover, these constitutive HSPs, along with *Hsp27*, were significantly more upregulated in Ganetespib-resistant than in sensitive tumors (Fig. 2a–c, *p* < 0.01). In contrast, UDP glucuronosyltransferase 1A (*UGT1A*), previously implicated in primary—not acquired—Ganetespib resistance in colorectal cancer^38^, was not upregulated (Fig. 2g). In agreement, mass spectrometry confirmed upregulation of inducible Hsp90, Hsp70 and Hsp27, as well as the endoplasmic reticulum (ER)-specific constitutive Hsp90 and Hsc70 at the protein level (Fig. 2h). Note, protein upregulation was evident at both 3 h and 24 h timepoints, i.e., lasted longer than the mRNA upregulation. These data point towards a broadly upregulated compensatory HSP program as the most upstream mechanism of acquired Ganetespib resistance.

### **U**pregulation of the phospho-RTKs/Akt signaling pathway in Ganetespib-resistant tumors

**Fig. 3:**
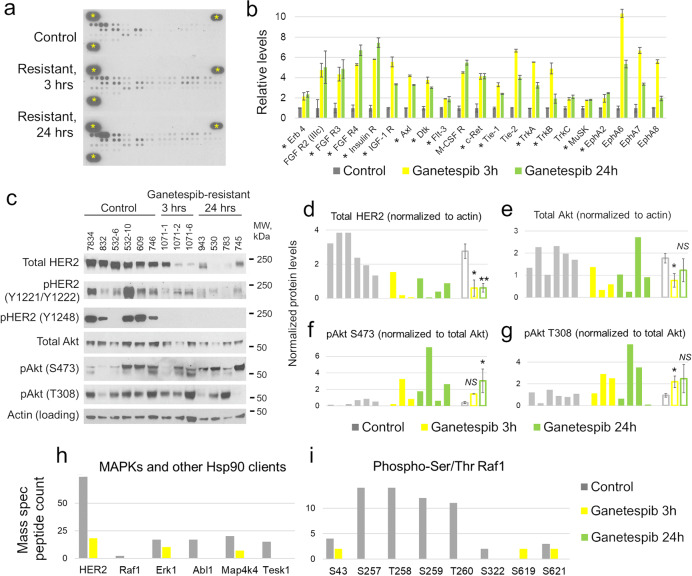
Activation of receptor tyrosine kinases (RTKs) and Akt/phospho-Akt in Ganetespib-resistant Neu tumors. **a**, **b** Phospho-kinome assay. **a** Representative immunoblots; yellow asterisks, reference markers. **b** Quantification of individual p-RTKs. Only the p-RTKs significantly upregulated at both timepoints (3 h, 24 h) in Ganetespib-resistant vs. Control samples are shown, i.e., 21 out of 39 p-RTKs. Mean ± SD of two technical replicas. Asterisks, the RTKs known as Hsp90 clients. **c**–**g** Western blots with the indicated antibodies (**c**) and their quantification by densitometry normalized as indicated (**d**–**g**). While HER2 is destabilized in Ganetespib-resistant tumors (**c**, **d**), its effector Akt is not (**c**, **e**) and is activated at two different phosphorylation sites, Ser 473 and Thr 308 (**c**, **f**, **g**). Empty bars are mean ± SEM of the tumors in each group. *p < 0.05, **p < 0.01, *NS*, not significant, unpaired two-tailed Student’s *t*-test. **h**, **i** Protein levels of HER2, its effectors Raf1, Erk1, and other known Hsp90 clients (**h**), and phospho-Ser/Thr Raf1 (**i**) by mass spectrometry. Note that the protein levels of HER2, Raf1, Erk1, p-Raf1 and known Hsp90 clients are all downregulated at both, 3 h and 24 h timepoints.

Many receptor tyrosine kinases (RTKs), besides HER2, are Hsp90 clients (www.picard.ch/downloads/Hsp90interactors.pdf) and also, frequently mediate therapeutic resistance, including in HER2-positive breast cancer^21,22,30^. Therefore, we analyzed a wide range of activated RTKs in a phospho-kinome assay on Ganetespib-resistant Neu tumor lysates. We found that 54% of all analyzed RTKs were significantly phospho-activated at both 3 h and 24 h after the last Ganetespib dose (21 out of 39), e.g., FGFRs, insulin receptor, insulin-like growth factor receptor, Tie-1/2, TrkA/B, EphA6-8 etc. (Fig. 3a, b). Note that many of them are known Hsp90 clients (Fig. 3b, asterisks). In addition, four RTKs were significantly phospho-activated only at 3 h and three RTKs only at 24 h, while three p-RTKs were significantly downregulated at 3 h, including p-HER2 (data not shown). These data indicate that a broad network of RTKs is stably activated in Ganetespib-resistant tumors.

RTKs, including HER2, signal via two major downstream pathways: the MAPK (Ras-Raf-Mek-Erk) pathway and the PI3K-Akt-mTOR pathway^14,22^. Therefore, we tested whether these pathways are involved in acquired Ganetespib resistance. First, we analyzed the levels of HER2 itself, which is a client of not only Hsp90A, but also Hsp70, Hsc70 and Hsp27^36,39,40^. We found that total HER2 and phospho-HER2 (Tyr 1248, Tyr 1221/1222) were still greatly suppressed in the resistant tumors at 3 h and 24 h after the last Ganetespib dose (Fig. 3c, d, h). Furthermore, the levels of HER2 effectors Erk1 and Raf1—also Hsp90 clients—were significantly reduced in the resistant tumors, as was phospho-Raf1 (Fig. 3h, i). As a positive control, known Hsp90 clients Abl1, Map4k4 and Tesk1 were also downregulated (Fig. 3h). In contrast, the levels of Akt/Protein kinase B—the other major RTK effector—were barely affected in Ganetespib-resistant tumors (Fig. 3c, e), even though Akt is a well-established Hsp90 client and therefore, is expected to be downregulated by Ganetespib^13,41^. Moreover, activated p-Akt, at Thr 308 and Ser 473, were significantly upregulated in the resistant tumors at 3 h and 24 h, respectively (Fig. 3c, f, g). Altogether, this suggests that Akt/p-Akt represents an essential signaling effector of the upregulated p-RTK network as a mechanism of acquired Ganetespib resistance.

### **P**harmacological inhibition of Akt significantly delays acquired Ganetespib resistance

**Fig. 4:**
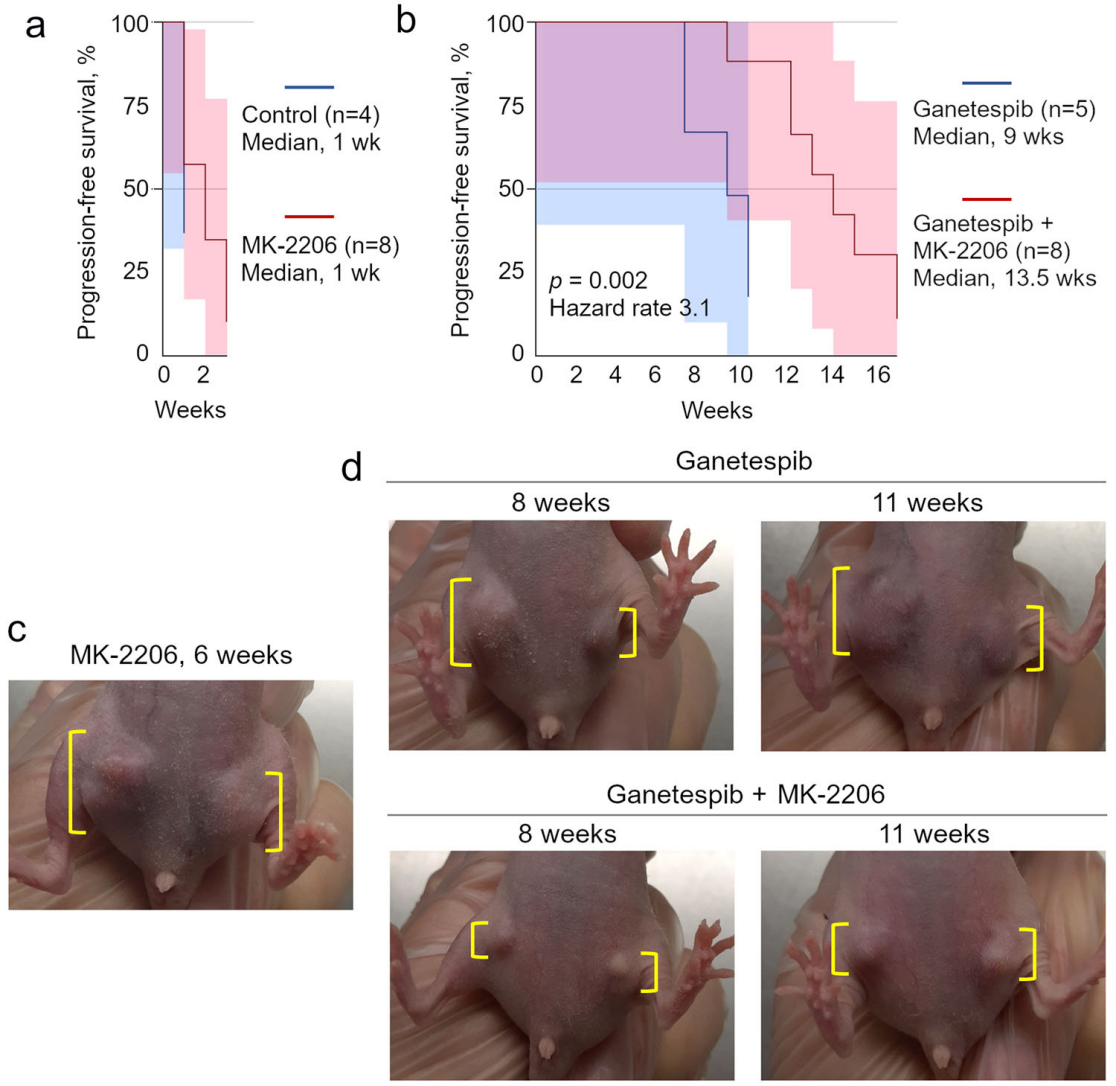
Akt inhibition significantly delays Ganetespib resistance in vivo. Immunodeficient female recipients were allografted with primary tumor cells from Neu mice into mammary fat pads and upon tumor onset, received either no drug (Control), a pan-Akt inhibitor MK-2206 alone, Ganetespib alone, or both (Ganetespib + MK-2206). The initial tumor size (when treatments started) was comparable for all four groups and was 87 ± 31 mm^3^, 88 ± 9 mm^3^, 90 ± 20 mm^3^, 91 ± 20 mm^3^ (mean ± SEM), respectively. **a**, **b** Progression-free survival comparing the indicated cohorts. Kaplan–Meier analysis, Log Rank statistics (*p*). Shaded areas are 95% confidence interval. **c**, **d** Representative tumors at the indicated time after treatment initiation. Note a significantly smaller tumor size in the mice treated with combined Ganetespib + MK-2206 compared with Ganetespib alone or MK-2206 alone (yellow brackets).

To test directly whether Akt/p-Akt mediates acquired Ganetespib resistance in vivo, we combined Ganetespib with a suboptimal dose of a pan-Akt inhibitor MK-2206^42,43^. MK-2206 is an orally bioavailable, highly selective, allosteric Akt1/2/3 inhibitor that—in contrast to ATP-competitive inhibitors—does not cause compensatory phospho-activation of Akt^44^, EGFR and HER2^45^. MK-2206 has been evaluated in 50 Phase I/II clinical trials, 13 of which are on breast cancer including eight on HER2-positive breast cancer. In combination with Trastuzumab or Lapatinib, MK-2206 showed favorable safety and efficacy in patients who progressed on HER2-targeted therapies^46–48^. Using allografts of primary Neu tumor cells into mammary fat pads of immunodeficient female recipients, we found that MK-2206 by itself did not significantly affect progression-free survival at the low dose we used (20 mg/kg three times a week; its half-life is 40 h^42,43^) (Fig. 4a, c). Ganetespib alone was effective for 9 weeks (median progression-free survival), however, its combination with MK-2206 delayed acquired resistance to a median of 13.5 weeks, i.e., by 50% (Fig. 4b, d). These results indicate that Akt indeed is a principal mediator of acquired Ganetespib resistance in vivo. Based on these data, we propose that clinically, co-targeting Akt together with Ganetespib may represent a therapeutically superior regimen for the treatment of HER2-positive breast cancer.

## Discussion

**Fig. 5:**
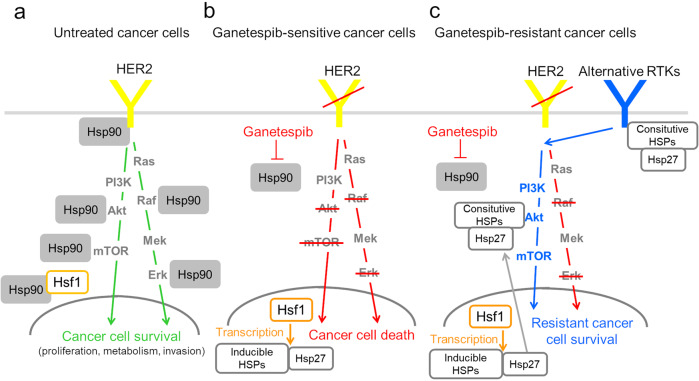
The proposed mechanism of acquired Ganetespib resistance in HER2-positive breast cancer. **a** In untreated HER2-positive breast cancer, HER2 and its signaling effectors, supported by the Hsp90 chaperone, promote the survival of cancer cells. **b** In the HER2-positive breast cancer cells sensitive to Ganetespib, Ganetespib blocks the oncogenic HER2 signaling pathway at multiple levels leading to tumor regression/stagnation. Ganetespib also stimulates the HSF1 transcription factor to activate inducible HSPs, e.g., Hsp27. **c** In the HER2-positive breast cancer cells resistant to Ganetespib, the upregulated HSPs, in particular Hsp90B, Hsc70 and Hsp27, stabilize multiple compensatory RTKs and their effector Akt/phospho-Akt, leading to tumor survival and growth. Therefore, Akt represents a central actionable node downstream of the HSP/p-RTK resistance program and a therapeutic vulnerability.

In this study we used autochthonous, immune-competent mouse models to investigate the mechanisms of acquired Ganetespib resistance in vivo. We have focused on HER2-positive breast cancer since it has proven to be highly sensitive to Hsp90 inhibition in the clinic^25–27^. We propose that in naïve HER2-positive breast cancer cells, Ganetespib is highly effective by curbing the Hsp90 support of HER2 itself, its co-receptor EGFR and the effectors Raf, Erk1, Akt, mTOR (Fig. 5a, b)^14^. However, Ganetespib eventually evokes acquired resistance by upregulating compensatory HSPs, especially constitutive Hsp90B and Hsc70, as well as inducible Hsp27 (Fig. 5c). In agreement, Hsp27 was previously implicated in the acquired resistance to Hsp90 inhibitor 17-AAG in hepatocellular carcinoma^49^. Although some activation of these HSPs is also observed in Ganetespib-sensitive controls (Fig. 2a–c), it is significantly stronger in the resistant tumors, suggesting an HSP transcriptome rewiring that is actively selected for in the resistant tumor cells. Furthermore, upregulation of wild-type and mutant RTKs is a frequent mechanism of therapeutic resistance in HER2-positive breast cancer^21,22^. Indeed, we find a wide range of RTKs to be phospho-activated in Ganetespib-resistant tumors (Fig. 3a, b) and propose that their stability and/or activity is actively supported by the upregulated HSPs (Fig. 5c). We did not directly test this, because (1) many RTKs are already established as HSP clients (www.picard.ch/downloads/Hsp90interactors.pdf; asterisks in Fig. 3b)^50,51^, (2) it would be technically challenging to simultaneously intercept several compensatory HSPs at once to test this idea, especially in our preferred in vivo system, and (3) we sought to find a druggable node downstream (not upstream) of activated p-RTKs as the most clinically impactful goal. To this end, we found that downstream of p-RTKs, the MAPK pathway is still effectively shut down in the resistant tumors (Fig. 3c, d, h, i). In contrast, the Akt pathway is active, as evident by stabilized and phospho-activated Akt (Fig. 3c, e–g) and, most importantly, by the sensitivity of the resistant tumors to pharmacological inhibition of Akt (Fig. 4).

Akt activation is one of the most frequent alterations in human cancers and is associated with enhanced tumor cell survival, proliferation, invasiveness, and poor patient outcomes in breast and other cancers^52–54^. Notably, Akt activation is also associated with resistance to chemo-, radiation and targeted therapies, including in HER2-positive breast cancer^19,20,22,55–61^. Akt is usually activated in cancer cells by gene amplification, activating mutations or loss of its inhibitor, tumor suppressor PTEN (phosphatase and tensin homolog)^52,55–57^. On the other hand, Akt is an Hsp90 client and would be expected to be destabilized by Ganetespib^13,41^. Our finding that, in contrast to HER2, Akt is not destabilized suggests that either Akt is less sensitive to Hsp90 inhibition than HER2, as previously reported^14,62–64^, or that Akt is actively stabilized in the resistant tumors by upregulated HSPs, or both. Supporting an active stabilization mechanism by Hsc70 and/or Hsp27, Akt was previously shown to be stabilized by Hsc70 and destabilized by Hsp70^65^ and also, activated by Hsp27, both directly and indirectly, via loss of PTEN^66–70^. Stabilized Akt is likely activated by the upregulated p-RTKs^14,22^. We did not test these mechanisms directly since it would be technically challenging to intercept all of the identified HSPs (≥3), p-RTKs (≥21) and/or their combinations simultaneously or one by one, especially in vivo. Although this is a limitation of our study, it does not lessen the identified critical role of Akt.

Here, we focused on HER2-positive breast cancer since it has emerged as one of the malignancies that can benefit from Hsp90 inhibition the most^25–27^. Of note, our findings are in agreement with a previous study implicating Akt, as well as the MAPK pathway, in acquired Ganetespib resistance in KRAS-mutant non-small-cell lung cancer^71,72^. In contrast, another study on triple-negative breast cancer rather found that the JAK-STAT pathway is involved in acquired Ganetespib resistance^73^. These differences may be explained by the different cancer types investigated, as well as by the fact that the aforementioned studies analyzed only cultured cells, but not genetic models^71–73^. While cell lines-based studies have their benefits, genetic mouse models present a unique opportunity to analyze drug resistance mechanisms in the native tumor microenvironment and in the presence of the functional immune system (known to mediate the effects of Hsp90 inhibitors^74–77^), which we made use of.

In sum, here we report that HER2-positive breast cancer, a malignancy highly sensitive to Hsp90 inhibition, acquires Ganetespib resistance by upregulating compensatory HSPs and the RTK-Akt pathway. Since pharmacological inhibition of Akt significantly delays acquired Ganetespib resistance, this establishes Akt as a unifying druggable node downstream of the widely activated HSP and RTK programs and suggests that Akt co-targeting together with Ganetespib may represent a therapeutically superior strategy for the treatment of HER2-positive breast cancer. In addition, our findings may prove valuable in other HER2-driven malignancies, e.g., colorectal, gastroesophageal and lung cancer^78–80^.

## Supplementary information

Mass spectrometry dataset
